# Supplementary material for: Efficient intracellular delivery of proteins by a multifunctional chimaeric peptide in vitro and in vivo
Source: Nat Commun. 2021 Aug 26;12:5131. doi: 10.1038/s41467-021-25448-z (PMC8390694; doi:10.1038/s41467-021-25448-z)
Supplement: Supplementary file 1 — Supplementary information [file 41467_2021_25448_MOESM1_ESM.pdf]

Supplementary Information for：

Efficient intracellular delivery of proteins by a multifunctional chimaeric peptide *in vitro* and *in vivo*

Siyuan Yu[1,2], Han Yang[1,2], Tingdong Li[1,2], Haifeng Pan[1,2], Shuling Ren[1], Guoxing Luo[1], Jinlu Jiang[1], Linqi Yu[1], Binbing Chen[1], Yali Zhang[1], Shaojuan Wang[1], Rui Tian[1], Tianying Zhang[1], Shiyin Zhang[1], Yixin Chen[1], Quan Yuan[1,*], Shengxiang Ge[1,*], Jun Zhang[1], and Ningshao Xia[1,*]

1: State Key Laboratory of Molecular Vaccinology and Molecular Diagnostics, National Institute of Diagnostics and Vaccine Development in Infectious Diseases, Collaborative Innovation Centers of Biological Products, School of Public Health, Xiamen University, Xiamen 361102, China.

***:** Corresponding author. Email: Ningshao Xia (nsxia@xmu.edu.cn), Shengxiang Ge (sxge@xmu.edu.cn), Quan Yuan (yuanquan@xmu.edu.cn), State Key Laboratory of Molecular Vaccinology and Molecular Diagnostics, Xiamen University, Xiamen 361102, People's Republic of China. Fax: (86)-05922181258.

2: These authors contributed equally to this work.

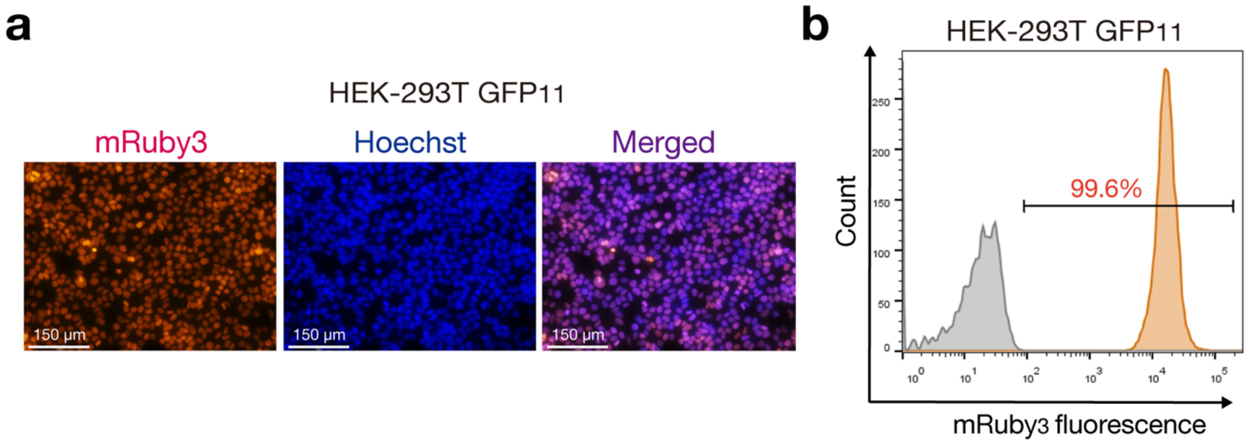

Supplementary figure. 1 The construction of HEK-293T-GFP$_{11}$ cell line. a Representative fluorescence microscopy images of HEK-293T GFP$_{11}$ cell line. Nucleus was stained with hoechst. The data shown are representative of three independent experiments. **b** The percentage of mRuby3 fluorescence-positive cells of HEK-293T-GFP$_{11}$ (orange) or wild type HEK-293T (grey) analyzed by FACS.

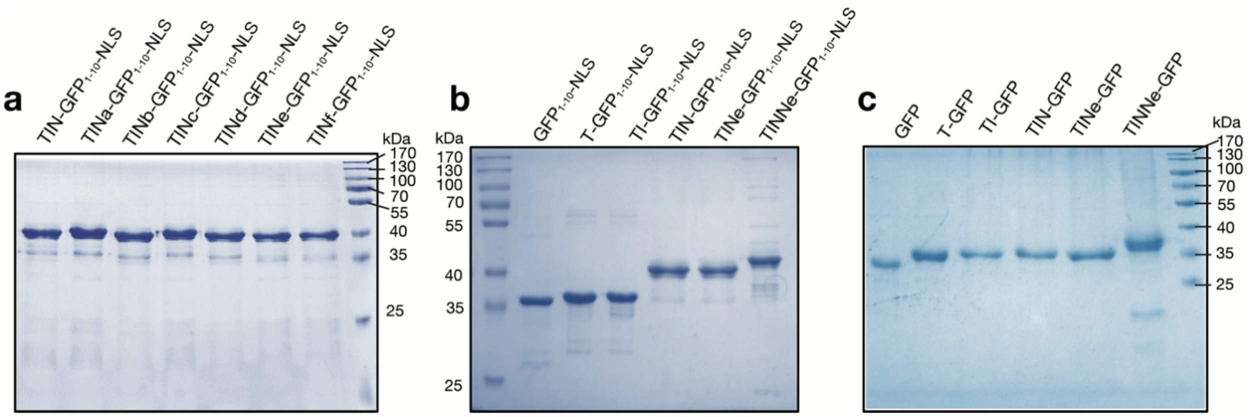

Supplementary figure. 2 The SDS-PAGE analysis of the GFP$_{1\text{-}10}$-NLS-related recombinant proteins (a, b) or GFP-related recombinant proteins (c). The sample of protein molecular weight markers was loaded in the first or last line of each cropped SDS-PAGE. The data shown are representative of two independent experiments respectively. The uncropped SDS-PAGEs are shown in Source Data File.

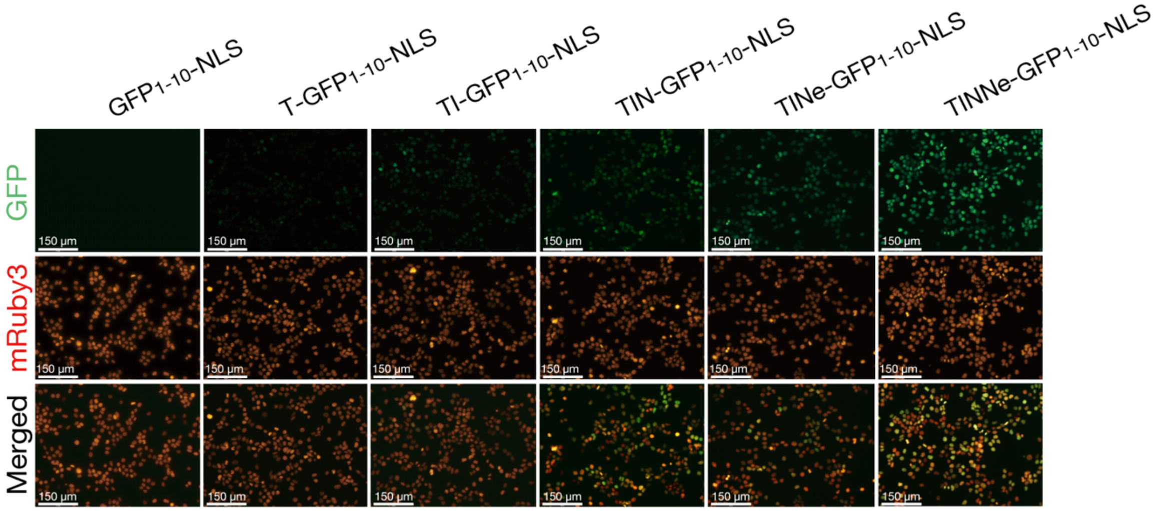

Supplementary figure. 3 Representative fluorescence microscopy images of the treated HEK-293T GFP$_{11}$ in Fig. 2d. The HEK-293T GFP$_{11}$ were incubated with GFP$_{1\text{-}10}$-NLS-related recombinant proteins (5 μM) for 3 h then washed, and imaged using fluorescence microscope at 12 h post incubation onset. The data shown are representative of three independent experiments.

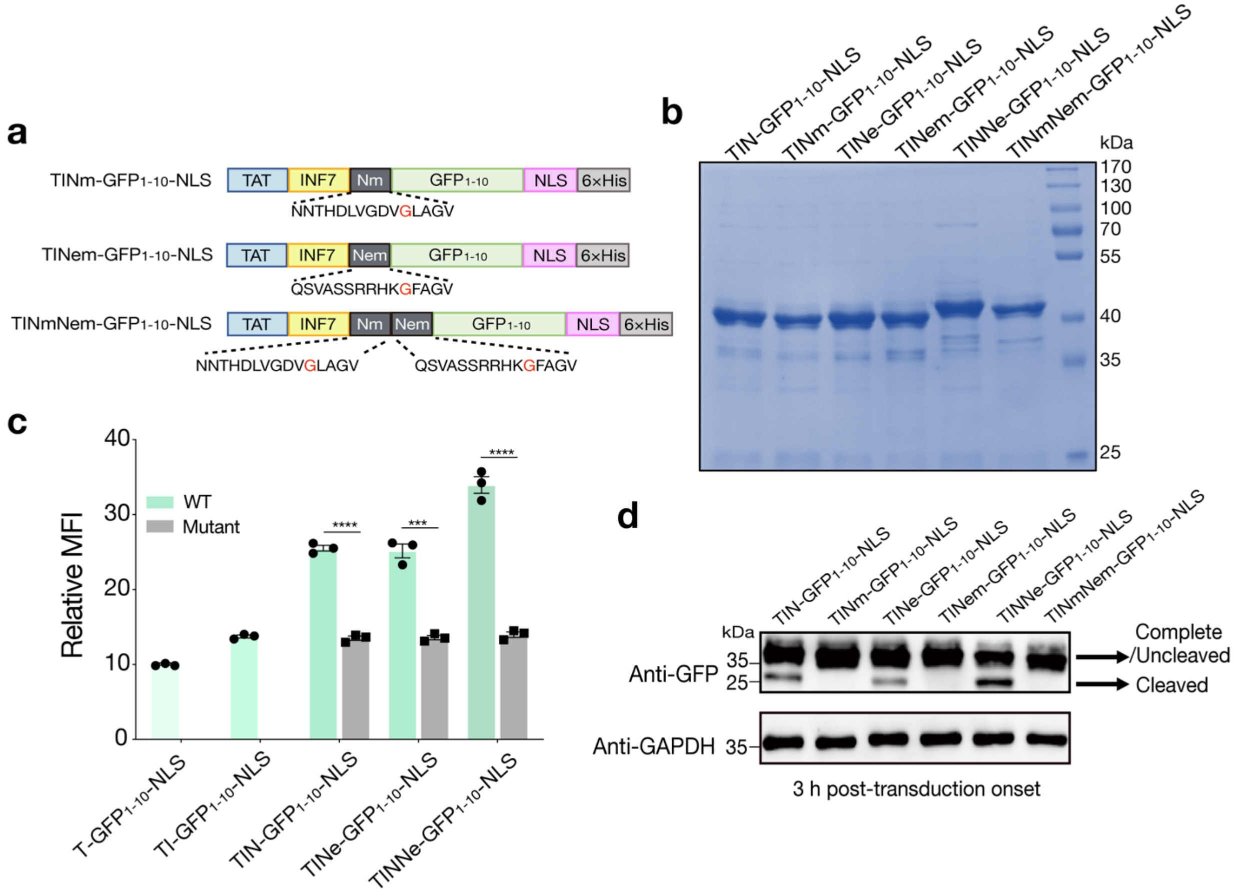

Supplementary figure. 4 Mutation of critical arginine residue to glycine in site N and Ne abrogates the cleavage in endosome and the improvement of endosomal escape. a Schematic diagram of different GFP_{1-10}-NLS related recombinant proteins. The amino acid sequence of mutant cleavage sites of N, Ne with critical arginine residue mutation (R>G) is presented below. **b** The SDS-PAGE analysis of GFP_{1-10}-NLS related recombinant proteins as indicated. The sample of protein molecular weight markers was loaded in the right line of cropped SDS-PAGE; the data shown are representative of two independent experiments. **c** The MFI of rescued GFP in treated HEK-293T GFP_{11} cells at end of endonuclear split-GFP assay. The GFP_{1-10}-NLS related proteins were used at a concentration of 5 μM. Results shown are means ± s.e.m.; n=3 biologically independent samples; *** $P < 0.001$, **** $P < 0.0001$; two-tailed unpaired student's t test. Relative MFI (fold increase) was obtained by MFI of total cells treated with the indicated proteins divided

by that of total cells treated with the corresponding cargo protein only. **d** Immunoblotting analysis in treated cells at 3 h post transduction onset during the process of endonuclear split-GFP assay. The $GFP_{1\text{-}10}$-NLS related proteins were used at a concentration of 1 μM. For **b**, the data shown are representative of three independent experiments; for **d** the data shown are representative of two independent experiments. For data, statistics, exact P-values, uncropped SDS-PAGE, and uncropped images of the immunoblots, see Source Data File.

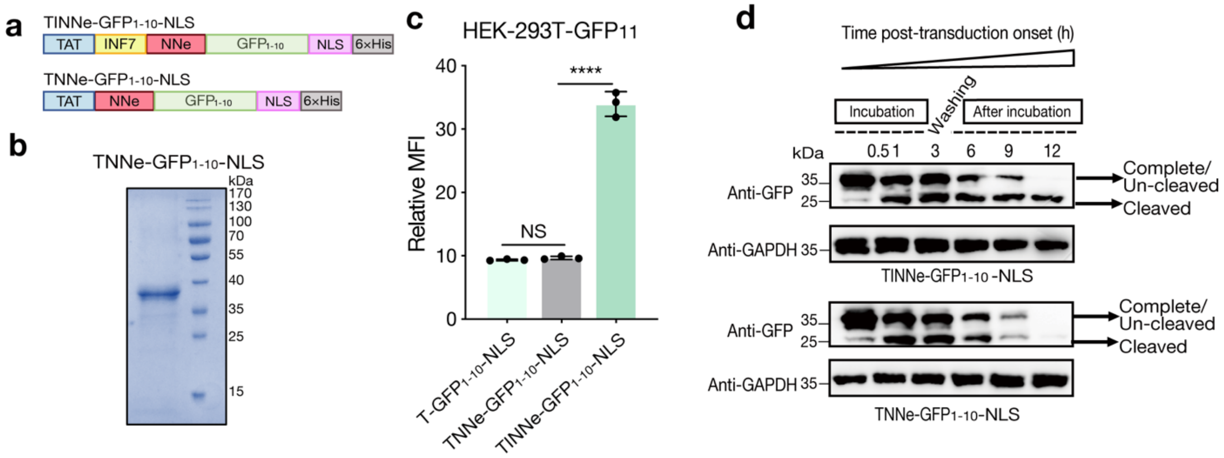

Supplementary figure. 5 Disruption of endosomal membrane by PMAP is the prerequisite for improvement of endosomal escape via proteolytic cleavage. **a** Schematic diagram of different GFP_{1-10}-NLS related recombinant proteins with or without PMAP(INF7). **b** The SDS-PAGE analysis of TNNe-GFP_{1-10}-NLS. The sample of protein molecular weight markers was loaded in the right line for cropped SDS-PAGE; the data shown are representative of three independent experiments. **c** The MFI of rescued GFP in treated HEK-293T GFP_{11} cells at end of endonuclear split-GFP assay. The GFP_{1-10}-NLS related proteins were used at a concentration of 5 μM. Results shown represents means ± s.e.m.; n=3 biologically independent samples; **** P<0.0001, NS, no significant difference; two-tailed unpaired student's t test. Fold was obtained by the MFI of a given protein in treated cells divided by the MFI of the GFP_{1-10}-NLS-treated cells. **d** Immunoblotting analysis in treated cells at different time point as indicated. The GFP_{1-10}-NLS related proteins were used at a concentration of 1 μM; the data shown are representative of two independent experiments. For data, statistics, exact P-values, uncropped SDS-PAGE, and uncropped images of the immunoblots, see Source Data File.

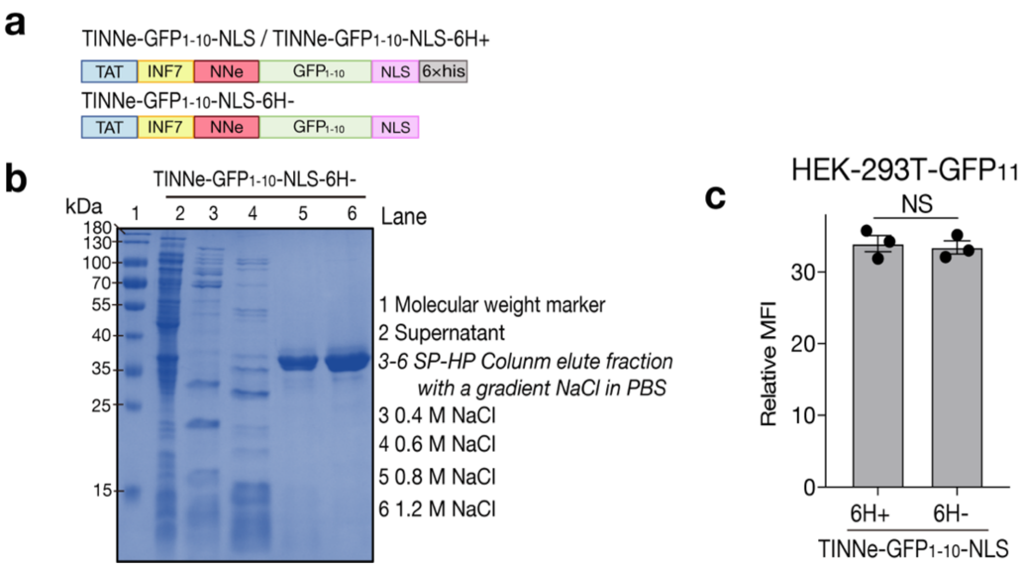

Supplementary figure. 6 The impact of fused-His-tag on efficiency of endosomal escape. a Schematic diagram of TINNe-GFP$_{1-10}$-NLS-6H- and TINNe-GFP$_{1-10}$-NLS-6H+. **b** SDS-PAGE analysis of TINNe-GFP$_{1-10}$-NLS-6H- at different purification steps. Sample in lane six was used for further experiment; the data shown are representative of two independent experiments. **c** The MFI of the HEK-293T cells treated with by 5 μM TINNe-GFP$_{1-10}$-NLS-6H- or TINNe-GFP$_{1-10}$-NLS-6H+. Results shown represent the means ± s.e.m.; n=3 biologically independent samples; NS, no significant difference; two-tailed unpaired student's t test. Fold was obtained by the MFI of a given protein in treated cells divided by the MFI of the GFP$_{1-10}$-NLS-treated cells. For data, statistics, exact P-values, and uncropped SDS-PAGE, see Source Data File.

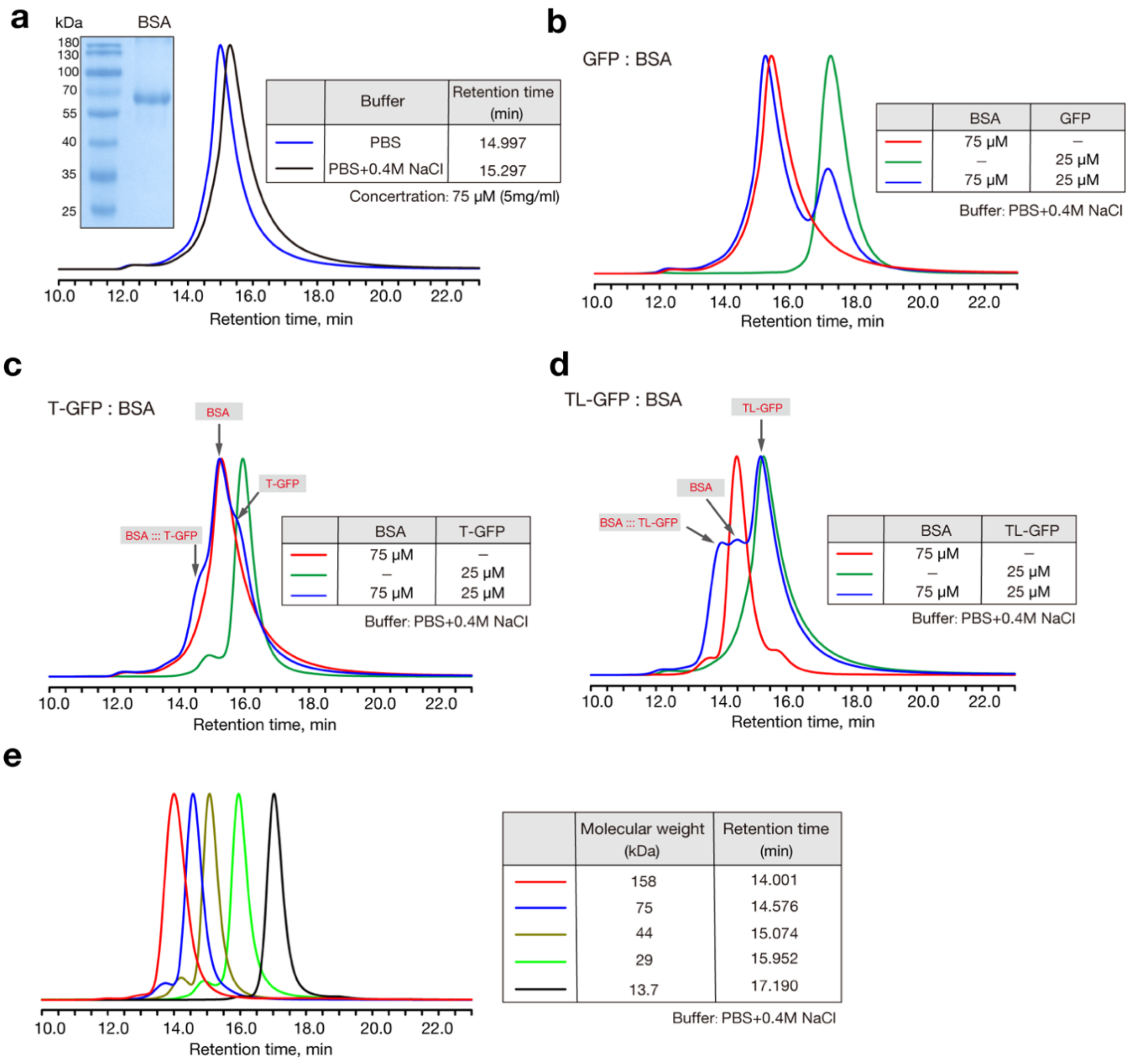

Supplementary figure. 7 The identification of the binding between TAT motif in the constructs with BSA. a The SDS-PAGE analysis of BSA (left panel), and the impact of buffer on retention time of BSA in HPSEC analysis; The sample of protein molecular weight markers was loaded in the left line for cropped SDS-PAGE. **b-d** The analysis of interaction between GFP-related proteins and BSA in the molar ratio of 1:3 using HPSEC**;** GFP **(b),** T-GFP **(c),** TL-GFP **(e). e** HPSEC retention time of protein molecular weight standards in the PBS+0.4 M NaCl. For **a** (left panel), the data shown are representative of three independent experiments. Uncropped SDS-PAGE is shown in Source Data File.

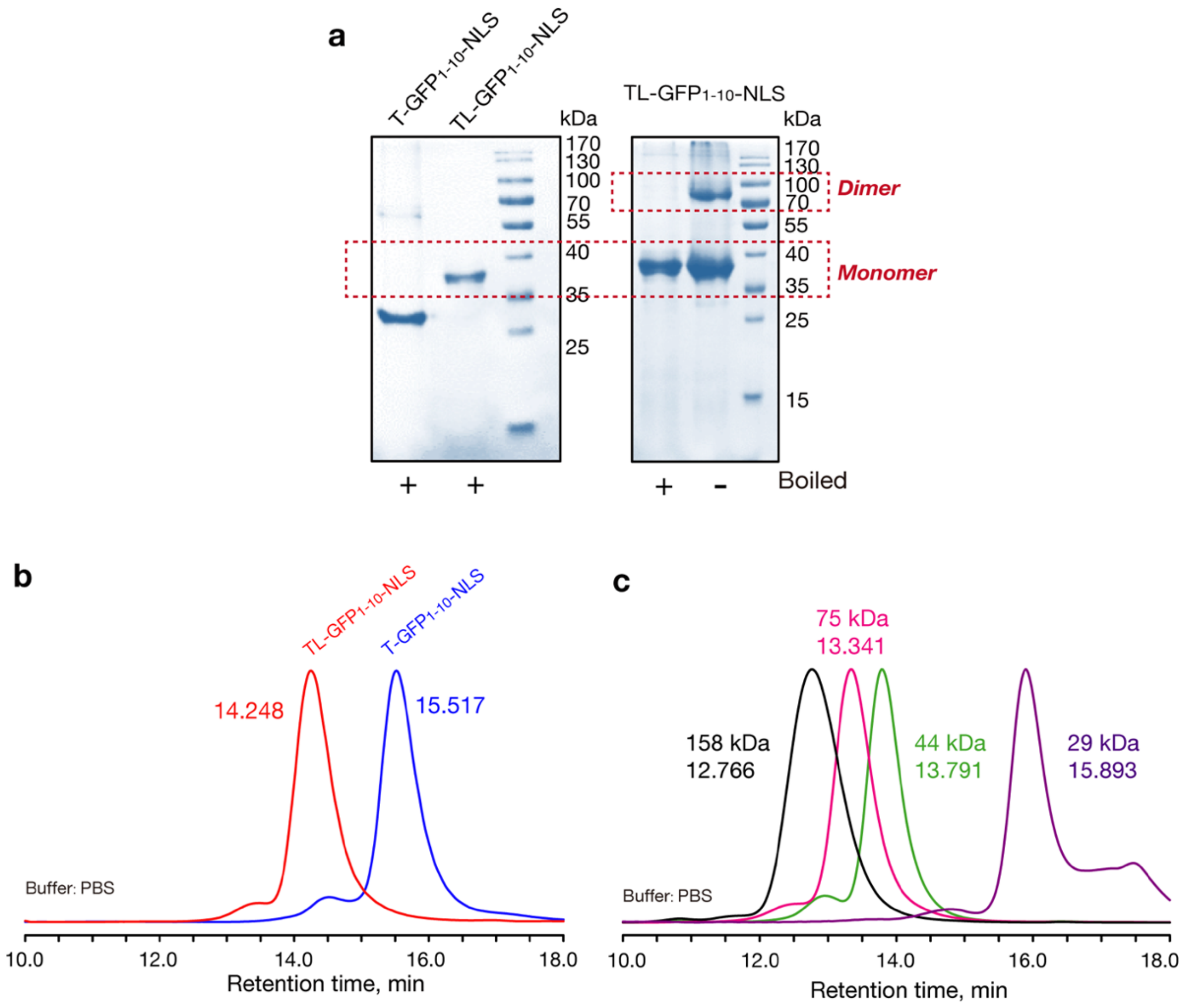

Supplementary figure. 8 The TAT-GFP$_{1\text{-}10}$-NLS and its counterpart containing leucine zipper were analyzed by reduced SDS-PAGE and HPSEC. a Samples in loading buffer were boiled (+) or not (-), and the sample of protein molecular weight markers was loaded in the right line for each cropped SDS-PAGE figure. **b** The HPSEC retention time of T- and TL-GFP in the PBS. **c** The HPSEC retention time of protein molecular weight standards in the PBS. For **a** (left and right panels**)**, the data shown are representative of three independent experiments respectively. Uncropped SDS-PAGEs are shown in Source Data File.

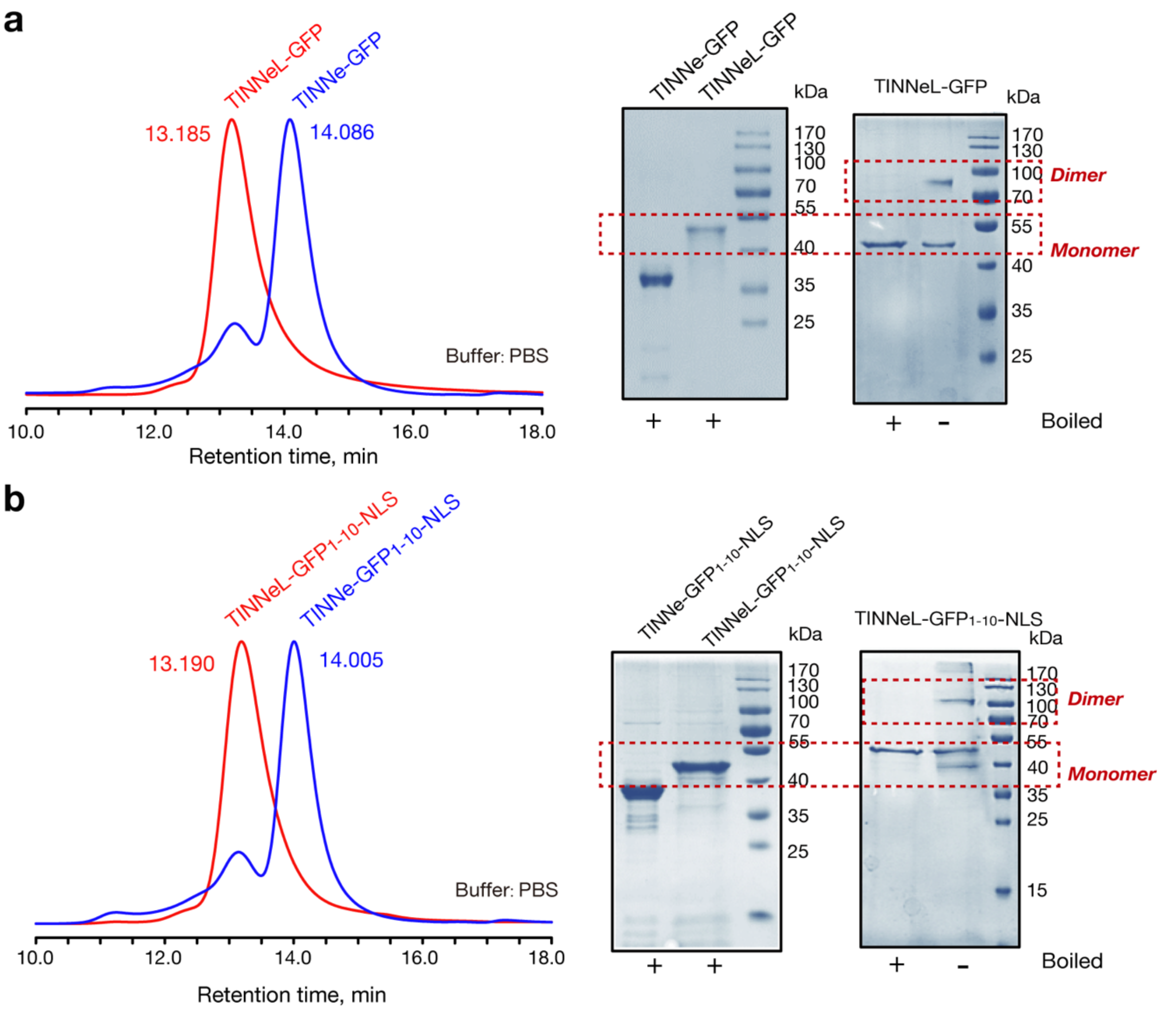

Supplementary figure. 9 The TINNe-GFP$_{1-10}$ and -GFP containing leucine zipper or not were analyzed by HPSEC (left panel) and reduced SDS-PAGE (right panel). a TINNe-GFP and TINNeL-GFP. **b** TINNe-GFP$_{1-10}$ and TINNeL-GFP$_{1-10}$. For SDS-PAGE analysis, protein samples of in loading buffer were boiled (+) or not (-), and the sample of protein molecular weight markers was loaded in the right line for each cropped SDS-PAGE figure. For SDS-PAGE analysis (right panels), the data shown are representative of three independent experiments respectively. Uncropped SDS-PAGEs are shown in Source Data File.

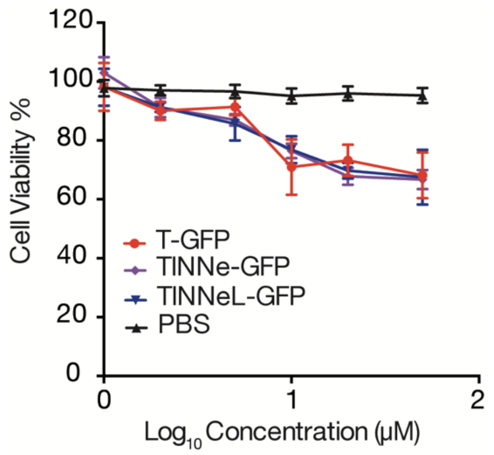

Supplementary figure. 10 The cytotoxicity of different GFP-related proteins. The HEK-293T cells were incubated with various concentrations (1, 2, 5, 10, 20, 50 or 100 μM) of T-, TINNe-, and TINNeL-GFP for 24 h, same volume of PBS was used as control. After incubation, cells were washed, and viability was analyzed by water-soluble tetrazolium-8 based cell counting kit-8 (CCK-8). Compared with T-GFP, TINNe-GFP and TINNeL-GFP did not show significant higher cytotoxicity. Results shown are mean ± s.e.m., n=3 biologically independent samples.

Quantitative determination of proteins delivered inside cells.

1. The establishment of standard curve for full length GFP and GFP_{1-10}-NLS

The standard curve for GFP was established by directly analyzing the fluorescence intensity of GFP protein of known concentration (Supplementary Fig.11a). As for the establishment of the standard curve for GFP_{1-10}-NLS, the progress curves for complementation of GFP_{1-10}-NLS and GFP_{11} in *vitro* was investigated firstly. We found that the fluorescence intensity starts to increase at 20 min after complementation starts, and reached plateau period at 4 hours (Supplementary Fig.11b). Accordingly, the standard curve of fluorescence intensity vs.GFP_{1-10}-NLS concentration was identified at 4 hours after complementation starts (Supplementary Fig.11c).

2. Quantitative determination of GFP inside cells

The HEK-293T were treated with 1 μM of GFP-related proteins in 1 mL serum-free DMEM for 3 h at 37 °C followed by the medium containing proteins was aspirated, and the cells were washed 3 times with DMEM containing 10 U/mL heparin. Cells were counted (1×10^5), harvested and lysed, then the amount of full-length GFP in lysate was quantified by measuring its fluorescence and converting to intracellular protein concertation using corresponding standard curve. Intracellular protein concertation was calculated as described in previous study(Erazo-Oliveras et al. (2014) Nat Methods, 11: 861-867.). We demonstrated that approximately 57.6 μM of GFP was present inside HEK-293T cells treated with TINNeL-GFP. Notably, consistent with the increment of MFI (fold increase) in FACS analysis, the amount of internalized cargo by TINNeL system was about 4-fold higher than that of TINNe and TAT peptide (Supplementary Fig.11d).

3. Quantitative determination of GFP_{1-10}-NLS in the nucleus

Likewise, HEK-293T-GFP_{11} were treated with 1μM GFP_{1-10}-NLS-related proteins in 1 mL serum-free DMEM for 3 h at 37 °C. At 12 h post incubation onset, the medium containing proteins was

aspirated, and the cells were washed 3 times with DMEM containing 10 U/mL heparin. Cells were counted (1×10^5 cells), harvested and lysed, then the amount of GFP_{1-10}-NLS in lysate was quantified as described in GFP section. Of note, considering the leakage of entrapped GFP_{1-10}-NLS from endosome during cell lysis would intervene the quantitative determination of GFP_{1-10}-NLS in the nucleus, all steps including cell lysis and the measurement of fluorescence emission intensity was accomplished within 10 min (*in vitro* fluorescence intensity starts to increase from 20 min post mixing) (Supplementary Fig.11b).On the basis of this assay, it was demonstrated that he amount of cytosolic delivery by TINNeL system is about 13-fold higher than that of TAT peptide only (Supplementary Fig.11e), and the similar increase was observed in the FACS analysis.

4. Identification of fraction of cytosolic delivery

Since the similar amount of internalization between GFP and GFP_{1-10}-NLS mediated by same delivery peptides (Supplementary Fig.11f), we provided objective quantitative insights into the process of TINNeL mediated-endosomal escape (the fraction of internalized cargo that reached the nucleus), which suggested that TINNeL induced at least 30% amount of total cellular internalization escaping from endosomes, remaining the same as TINNe, while only 10 % in that of T-GFP_{1-10}-NLS (Supplementary Fig.11g). This indicates that in comparison with TINNe, TINNeL mediated-higher level of absolute amount of escaping-cargo mainly relies on enhancement of total internalization induced by its Leu-zipper module.

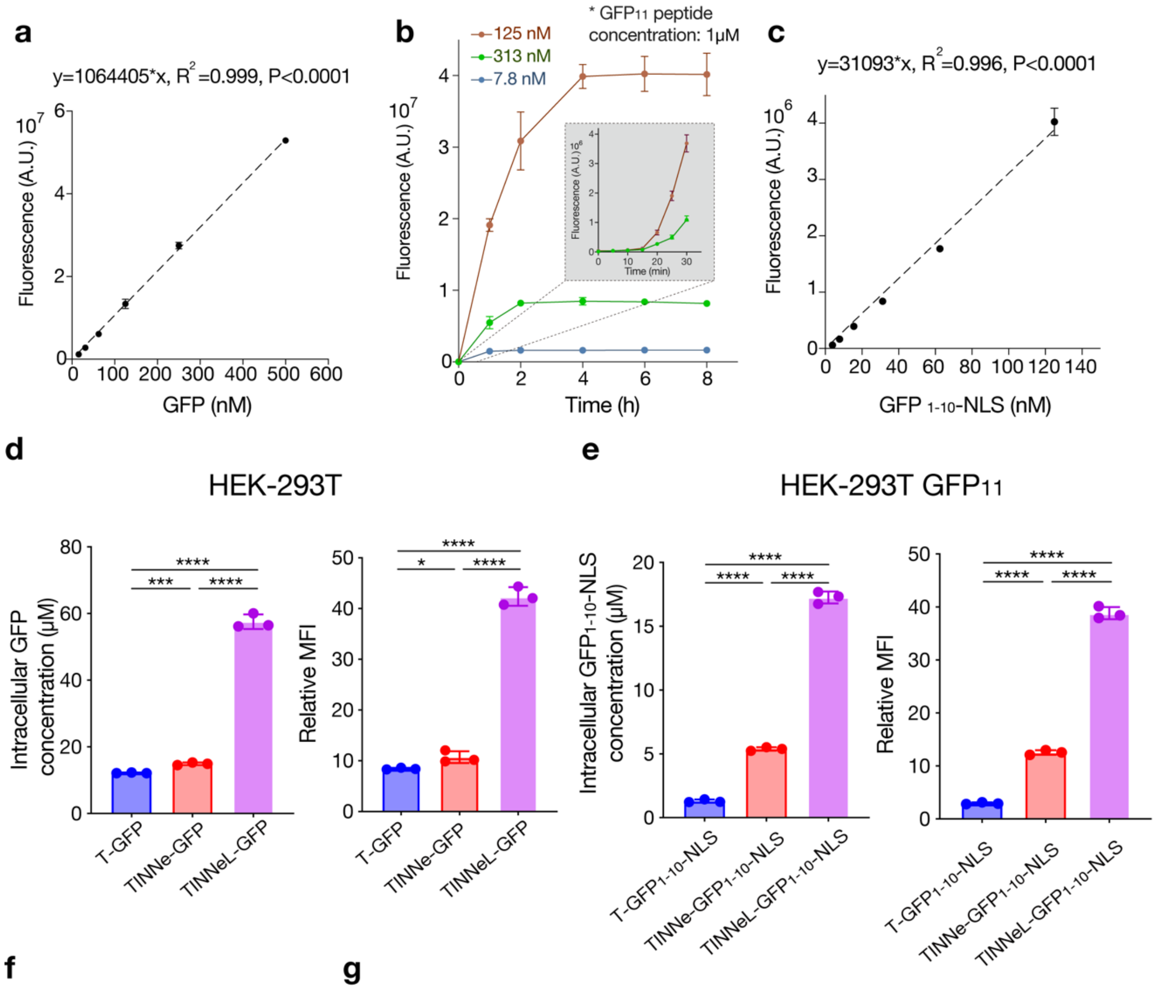

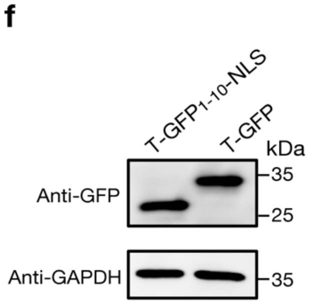

g

Delivery system	Cargo protein	Fluorescence intensity of cell lysate ($\times 10^6$A.U.)	Estimated intracellular protein concentration (μM) *	The fraction of cytosolic delivery (%)
**TAT**	Full length GFP	2.59	12.15	**10.6**
	GFP1-10-NLS	0.81	1.30	
**TINNe**	Full length GFP	3.17	14.90	**33.9**
	GFP1-10-NLS	3.18	5.05	
**TINNeL**	Full length GFP	11.82	55.50	**30.3**
	GFP1-10-NLS	10.73	17.05	

*:Number of cells contained in the lysate: 1×10^5 ; Estimated volume of one cell: 2×10^{-12}L
All experiments were performed in triplicate.

Supplementary figure. 11 The determination of absolute amount of protein delivered into cells or in the nucleus mediated by various delivery system. **a** The standard curve of fluorescence intensity vs.GFP concentration was established. The fluorescence emission intensity of 100 μL aliquots containing 1.56 to 50 pmol of GFP were measured. **b** Progress curves for complementation of GFP1-10-NLS of known concentration as indicated. 50 μL aliquots containing

0.78, 3.13 and 12.5 pmol of GFP_{1-10}-NLS were mixed with 50 μL aliquots containing 100 pmol GFP_{11} peptides to start complementation, and fluorescence emission intensity was measured at indicated time point. **c** The standard curve of fluorescence intensity vs.GFP_{1-10}-NLS concentration was established. 50 μL aliquots containing 0.39 to 12.5 pmol of GFP_{1-10}-NLS were mixed with 50 μL aliquots containing 100 pmol GFP_{11} peptides to start complementation, and fluorescence emission intensity was measured at 4 h post mixing. **d** Intracellular GFP concentration (left panel) and relative MFI of HEK-293T cells treated by 1 μM T-, TINNe-, or TINNeL-GFP in serum-free DMEM (1 mL). **e** Intracellular GFP_{1-10}-NLS concentration (left panel) and relative MFI of HEK-293T GFP_{11} cells treated 1 μM T-, TINNe-, and TINNeL-GFP_{1-10}-NLS in serum-free DMEM (1 mL). **f** Immunoblot analysis of level of T-GFP and T-GFP_{1-10}-NLS (1 μM); the data shown are representative of two independent experiments. **g** The identification of the fraction of internalized cargo that reached the nucleus based on various delivery systems. Results in **a-e** shown are means ± s.e.m.; n = 3 for each group. For d and e, two-tailed unpaired student's t test; * $P < 0.05$, *** $P < 0.001$, **** $P < 0.0001$, and NS, no significant difference. For data, statistics, exact P-values, and uncropped images of the immunoblots, see Source Data File.

a

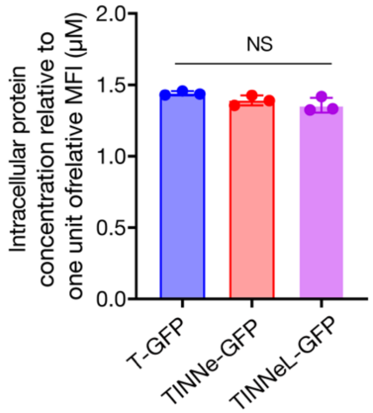

Constructs	Relative MFI analyzed by FACS (fold increase)	Estimated Intracellular protein concentration (μM)	Intracellular concentration relative to one unit of relative MFI (μM)
**T-GFP**	8.45	12.15	**1.44**
**TINNe-GFP**	10.72	14.90	**1.39**
**TINNeL-GFP**	42.39	55.50	**1.36**

b

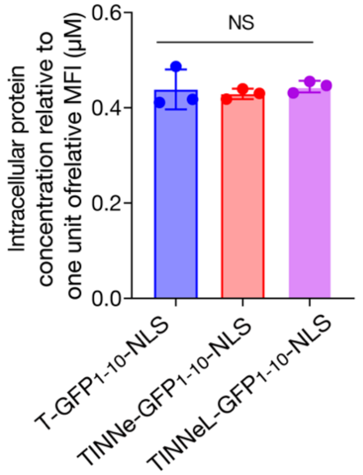

Constructs	Relative MFI analyzed by FACS (fold increase)	Estimated Intracellular protein concentration (μM)	Intracellular concentration relative to one unit of relative MFI (μM)
**T-GFP$_{1-10}$-NLS**	2.96	1.30	**0.44**
**TINNe-GFP$_{1-10}$-NLS**	12.55	5.05	**0.43**
**TINNeL-GFP$_{1-10}$-NLS**	38.93	17.05	**0.44**

Supplementary figure. 12 The identification of intracellular GFP concentration(a) or GFP$_{1-10}$-NLS concentration(b) relative to one unit of relative MFI. The value of each dot was obtained by that intracellular protein concentration divided by its corresponding relative MFI analyzed by FACS. Results shown in **a-b** are means ± s.e.m., n = 3 for each group. NS, no significant difference; two-tailed unpaired student's t test. For data, statistics, and exact P-values, see Source Data File.

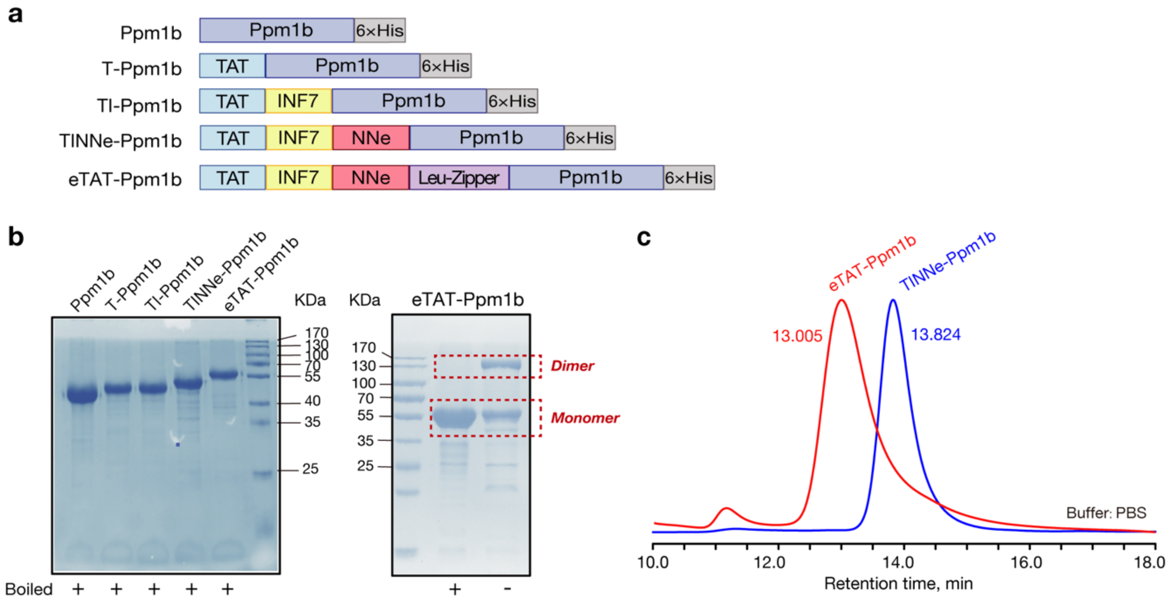

Supplementary figure. 13 Recombinant Ppm1b protein and Ppm1b fusion proteins. **a** Scheme of different Ppm1b related-recombinant proteins used in Fig.3 and 4. **b** Reduced SDS-PAGE analysis of recombinant Ppm1b and Ppm1b fusion proteins. Samples in loading buffer were boiled (+) or not (-), and the sample of protein molecular weight markers was loaded in the first or last line for each cropped SDS-PAGE. **c** HPSEC analysis of TINNe- and eTAT-Ppm1b. For **b**, the data shown are representative of three independent experiments respectively. Uncropped SDS-PAGEs are shown in Source Data File.

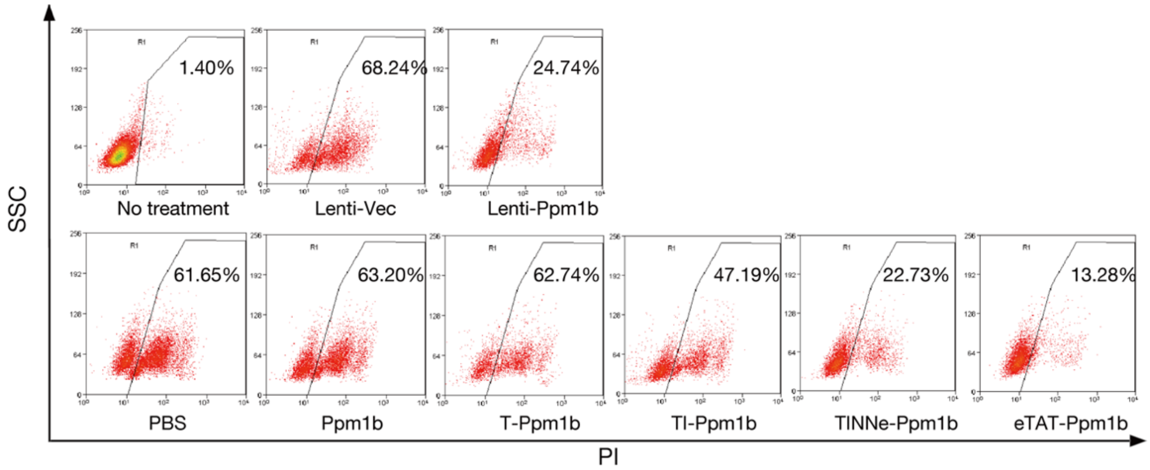

Supplementary figure. 14 Representative flow cytometry of L929 incubated with Ppm1b related-recombinant proteins in Figure 3b (upper panel). The percentages of PI^+ cells are indicated.

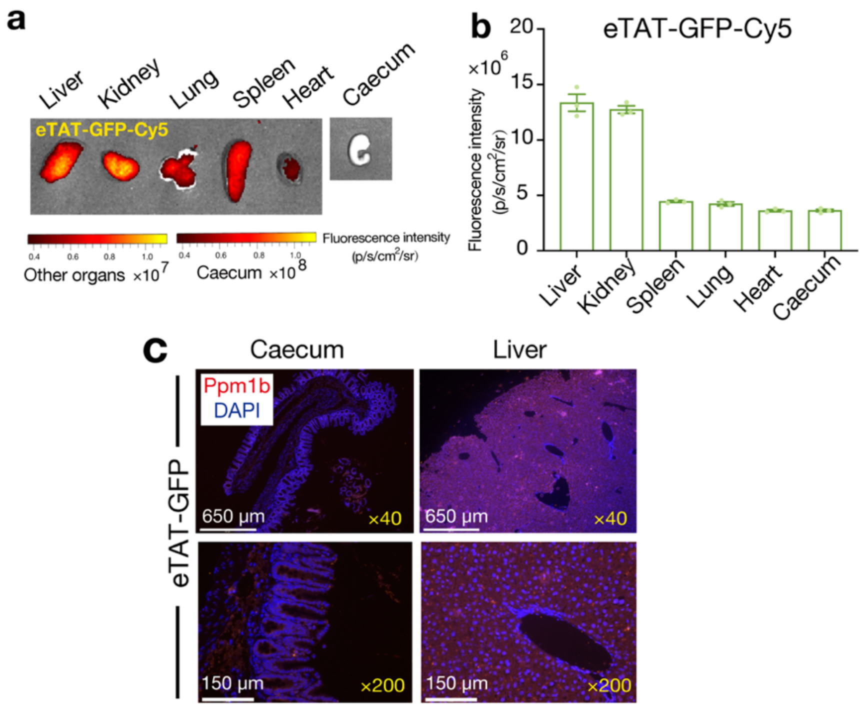

Supplementary figure. 15 *In vivo* protein delivery of eTAT-GFP. a, b Distribution of intravenously administered Cy5 labelled-eTAT-GFP in the female BALB/c mice. Representative microscope images **(a)** and florescence intensity **(b)** in the indicated organs of the BALB/c mice intravenously administered with eTAT-Ppm1b-Cy5 (n=3). Twenty-four hours post-administration, the mice were sacrificed, and fluorescence imaging of each organ was performed. **c** Five hours after intravenous injection of eTAT-GFP, the tissues were harvested and prepared as slides. The nuclei were stained with DAPI, and the delivered Ppm1b were detected using anti-6×his IgG as the primary antibody and Alexa 647-conjugated anti-mouse IgG as the secondary antibody. The fluorescence was observed and imaged via fluorescence microscopy. Results in **b** shown are mean ± s.e.m., n =3 mice for each group. For **c**, the data shown are representative of three independent experiments respectively.

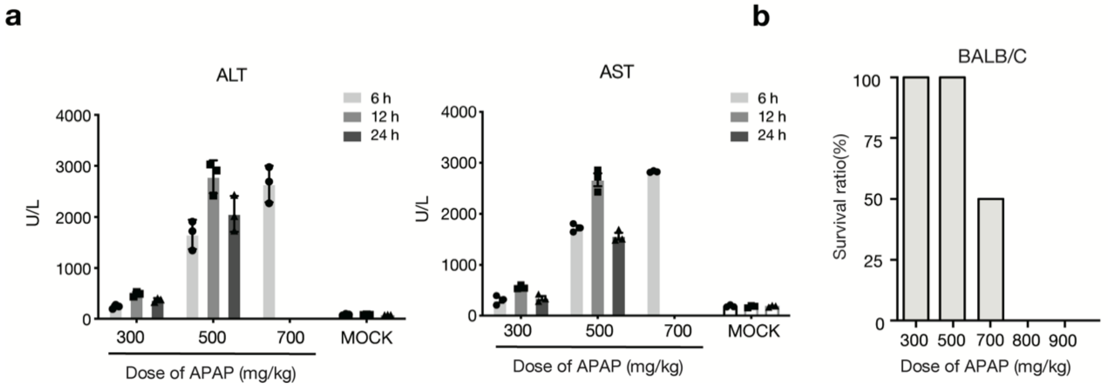

Supplementary figure. 16 Identification of the (a) maximum non-lethal and (b) minimum absolute lethal doses of APAP in female BALB/C. **a** Wild-type female BALB/c mice were treated intravenously with 300, 500 or 700 mg/kg acetaminophen, serum was collected at 6, 12 and 24 h post APAP administration, then activities of ALT and AST were determined by routine clinical assays using commercial kits (At 12 h, two mice in the dose of 700 mg/kg were died, so the ALT/AST level were not shown). The mock mice did not receive any reagent. The highest dose of APAP that induced obvious increasement of ALT and AST, while did not kill mice, 500 mg/kg, was taken as the maximum non-lethal doses in our subsequent researches. Results shown are means ± s.e.m., n = 3 mice for each group. **b** Wild-type BALB/C were injected intravenously with different dose of APAP (300, 500, 700, 800, 900 mg/kg). Then mouse survival was monitored during 48 h post challenge. Graph shows total mouse survival at 24 h after APAP intoxication. n = 8 mice for each group. The lowest dose of APAP that killed all mice, 800 mg/kg, was taken as the minimum absolute lethal doses in our subsequent researches.

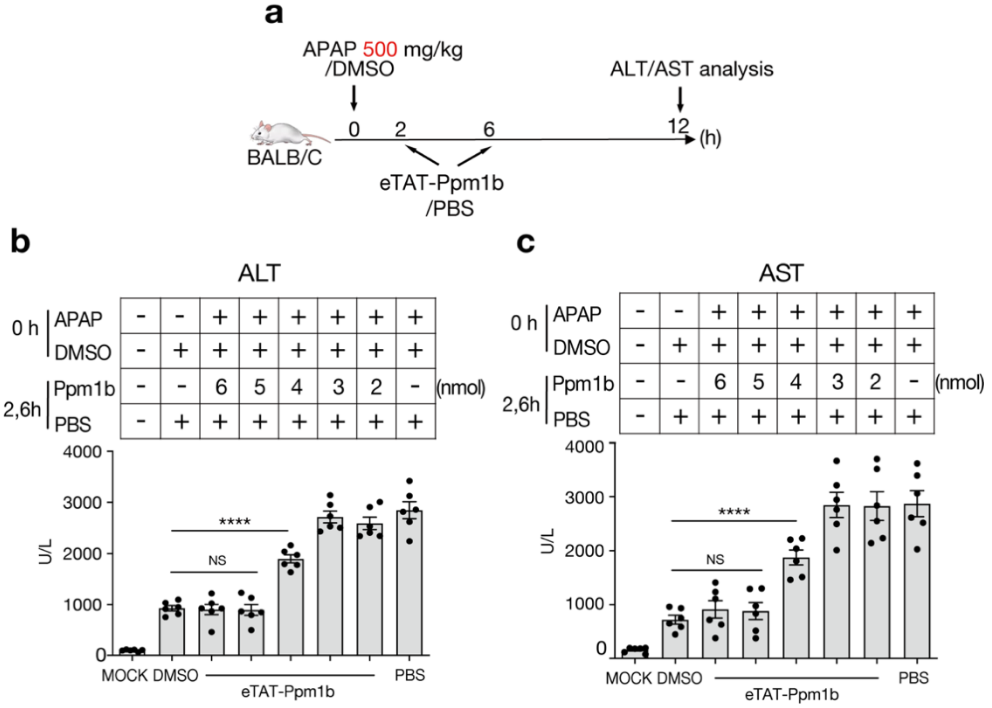

Supplementary figure. 17 Identification the minimum dose of eTAT-Ppm1b required to achieve therapeutic effect in female BALB/C. **a** Wild-type female BALB/c mice were intravenously administrated with 2 to 6 nmol eTAT-Ppm1b twice in 2 h and 6 h post-APAP administration. **b-c** Serum was collected at 12 h post APAP administration, then activities of **(b)** ALT and **(c)** AST were determined by routine clinical assays using commercial kits**.** Results in **b-c** (bottom panels) shown are means ± s.e.m.; n = 7 mice for each group; **** P<0.0001, and NS, no significant difference; two-tailed unpaired student's t test. For data, statistics, and exact P-values, see Source Data File.

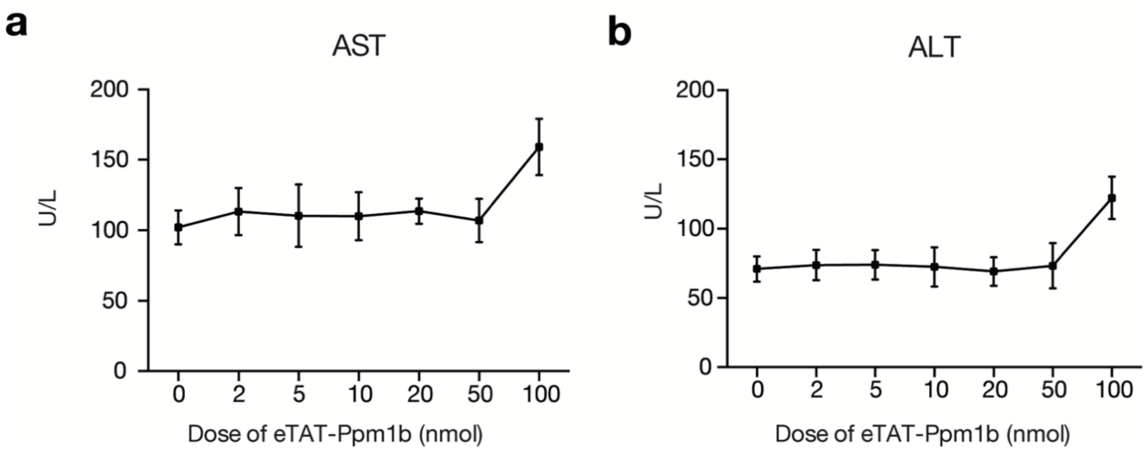

Supplementary figure. 18 The *in vivo* toxicity of different dose of eTAT-Ppm1b. Wild-type BALB/c mice were treated intravenously with various dose of eTAT-Ppm1b (i.v.), serum was collected at 12 h post treatment, and activities of **(a)** ALT, **(b)** AST were determined by routine clinical assays using commercial kits. Results shown are means ± s.e.m, n=3 mice for each group.

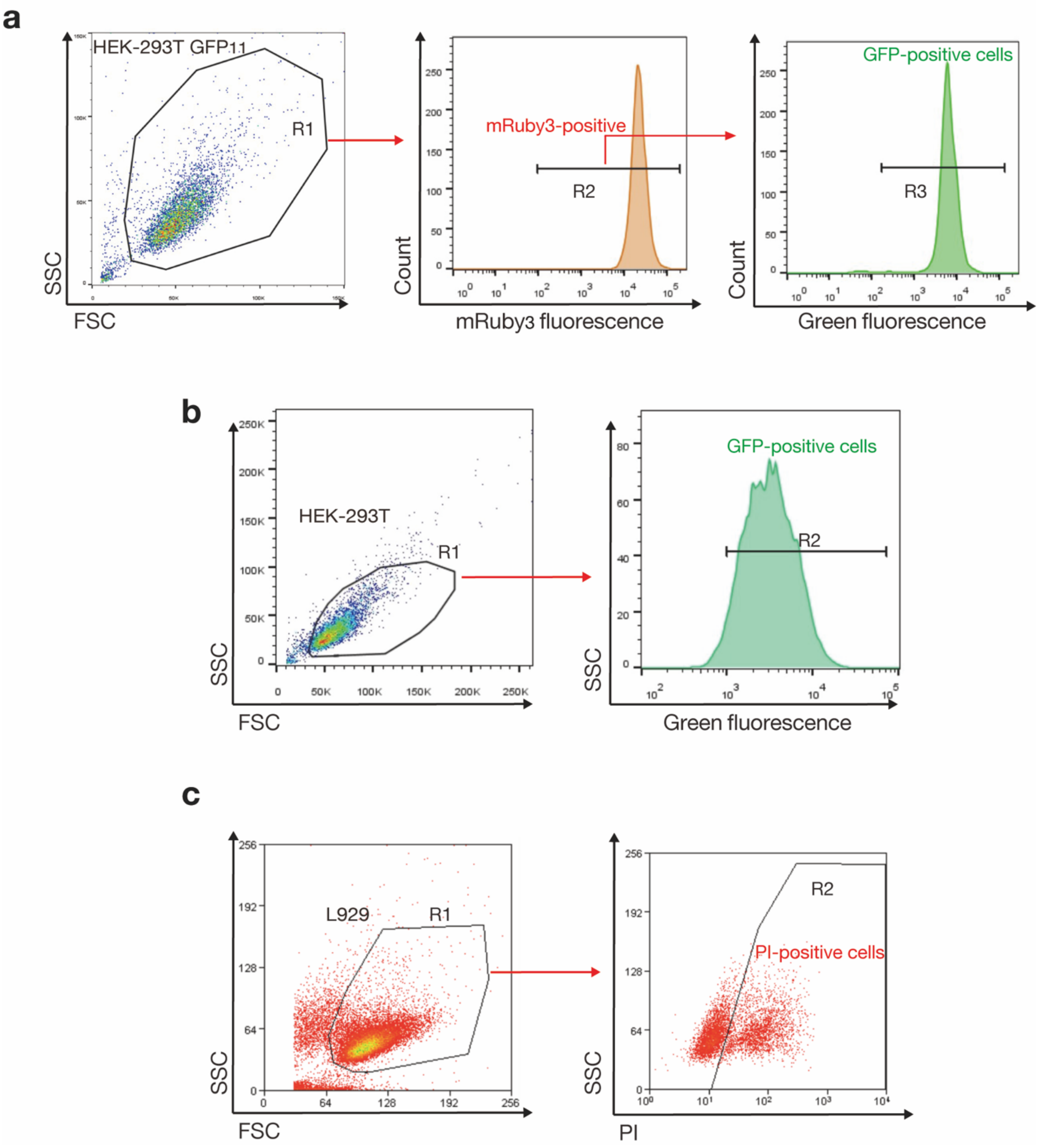

Supplementary figure. 19 Gating strategies used for cell sorting. a Gating strategy to sort GFP-positive cells in HEK-293T-GFP$_{11}$ treated with GFP$_{1-10}$-NLS-related recombinant proteins. The cells displaying a normal morphology were gated first (left panel, R1); these cells expressing mRuby3 were gated (middle panel, R2); then their GFP fluorescence was analyzed (right panel, R3). The calculated mean fluorescent intensity within total mRuby3-fluorescence-positive cells and the percentage of green fluorescence-positive cells were recorded. This strategy was used on

the analysis of delivery efficiency presented in the Figs 1c, 1d, 1f, 2d, 2i, 2k, and Supplementary figures 1b, 4c, 5c, 6c, 11e (right panel). **b** Gating strategy to sort GFP-positive cells in HEK-293T treated with GFP-related recombinant proteins. The cells displaying a normal morphology were gated first (left panel, R1); then their GFP fluorescence was analyzed (right panel, R2). The calculated mean fluorescent intensity within total HEK-293T cells and the percentage of green fluorescence-positive cells were recorded. This strategy was used on the analysis of delivery efficiency presented in the Figs 1h, 2a, 2d (right panel), 2e-g, and Supplementary figures 1b, 4c, 5c, 6c, 11d (right panel)**.** **c** Gating strategy to sort PI-positive cells in L929 treated with Ppm1b-related recombinant proteins. The cells displaying a normal morphology were gated first (left panel, R1); then their PI fluorescence (PE channel) was analyzed (right panel, R2). PI-positive cells were considered to be dead cells. This strategy was used on the analysis of cell death presented in the Fig 3b, and Supplementary figure.14.
