# Supplementary material for: Efficient intracellular delivery of proteins by a multifunctional chimaeric peptide in vitro and in vivo
Source: Nat Commun. 2021 Aug 26;12:5131. doi: 10.1038/s41467-021-25448-z (PMC8390694; doi:10.1038/s41467-021-25448-z)
Supplement: Supplementary file 3 — Reporting Summary [file 41467_2021_25448_MOESM3_ESM.pdf]

nature research

Corresponding author(s): Ningshao Xia, Shengxiang Ge and Quan Yuan

Last updated by author(s): Jul 28, 2021

Reporting Summary

Nature Research wishes to improve the reproducibility of the work that we publish. This form provides structure for consistency and transparency in reporting. For further information on Nature Research policies, see our Editorial Policies and the Editorial Policy Checklist.

Statistics

For all statistical analyses, confirm that the following items are present in the figure legend, table legend, main text, or Methods section.

n/a	Confirmed	
☐	☒	The exact sample size (n) for each experimental group/condition, given as a discrete number and unit of measurement
☐	☒	A statement on whether measurements were taken from distinct samples or whether the same sample was measured repeatedly
☐	☒	The statistical test(s) used AND whether they are one- or two-sided<br>*Only common tests should be described solely by name; describe more complex techniques in the Methods section.*
☒	☐	A description of all covariates tested
☐	☒	A description of any assumptions or corrections, such as tests of normality and adjustment for multiple comparisons
☐	☒	A full description of the statistical parameters including central tendency (e.g. means) or other basic estimates (e.g. regression coefficient) AND variation (e.g. standard deviation) or associated estimates of uncertainty (e.g. confidence intervals)
☐	☒	For null hypothesis testing, the test statistic (e.g. F, t, r) with confidence intervals, effect sizes, degrees of freedom and P value noted<br>*Give P values as exact values whenever suitable.*
☒	☐	For Bayesian analysis, information on the choice of priors and Markov chain Monte Carlo settings
☒	☐	For hierarchical and complex designs, identification of the appropriate level for tests and full reporting of outcomes
☒	☐	Estimates of effect sizes (e.g. Cohen's d, Pearson's r), indicating how they were calculated

Our web collection on statistics for biologists contains articles on many of the points above.

Software and code

Policy information about availability of computer code

Data collection	FACS: Summit v4.3, BD FACSDiva Software v9.0.<br>Fluorescence image: NIS-Elements F 4.00.00 (Build 764) version 4.0, Invitrogen EVOS M7000 Imaging System.<br>Western blotting: ImageQuant LAS 4000.<br>Histologic image: CellSens Standard 2010.<br>Cytotoxicity assay: Skanlt RE 6.0.1.
Data analysis	Summit v4.3 software; FlowJo X 10.0.7; Microsoft Excel 2013; Graphpad Prism software 6.0 & 7.0.

For manuscripts utilizing custom algorithms or software that are central to the research but not yet described in published literature, software must be made available to editors and reviewers. We strongly encourage code deposition in a community repository (e.g. GitHub). See the Nature Research guidelines for submitting code & software for further information.

Data

Policy information about availability of data

All manuscripts must include a data availability statement. This statement should provide the following information, where applicable:

- Accession codes, unique identifiers, or web links for publicly available datasets
- A list of figures that have associated raw data
- A description of any restrictions on data availability

All data supporting this study are available in the main figures and the Supplementary information files. Source data underlies the graphs in figures and Supplementary information are provided with the paper in a single excel file.

Field-specific reporting

Please select the one below that is the best fit for your research. If you are not sure, read the appropriate sections before making your selection.

- [x] Life sciences
- [ ] Behavioural & social sciences
- [ ] Ecological, evolutionary & environmental sciences

For a reference copy of the document with all sections, see nature.com/documents/nr-reporting-summary-flat.pdf

Life sciences study design

All studies must disclose on these points even when the disclosure is negative.

Sample size	All experiments established experimental group sizes from previous work were used to approximate sample sizes (Wadia JS et al. Nature Medicine;10, 310-315 (2004); Erazo-Oliveras A et al. Nature Methods;11, 861-867 (2014);Chen W et al. Nature Cell Biology;17, 434-444 (2015)).
Data exclusions	No data were excluded from analysis.
Replication	All experiments were repeated at least two times to confirm the reproducibility of the results.
Randomization	All samples were randomly assigned to experimental groups:for in vitro analyses on cultured cells, cells from same cultures were divided into different conditions; for in vivo analyses, mice of equal age were randomly assigned to the different conditions.
Blinding	For in vitro analyses: analysis of flow cytometry did not involve manual data selection, and was automated as far as possible to avoid potential biases; evaluation for fluorescence intensities from microscopic analysis was conducted in a blind manner; for western blotting, cells treated with different recombinant proteins were known when prepare the samples or set up the assay by one investigator, then data was analyzed by other blinded-investigators.<br>For in vivo analyses: investigators were blinded to the allocation during performing the histology experiments and scoring the tissue damage.

Reporting for specific materials, systems and methods

We require information from authors about some types of materials, experimental systems and methods used in many studies. Here, indicate whether each material, system or method listed is relevant to your study. If you are not sure if a list item applies to your research, read the appropriate section before selecting a response.

Materials & experimental systems

n/a	Involved in the study	
[ ]	[x]	Antibodies
[ ]	[x]	Eukaryotic cell lines
[x]	[ ]	Palaeontology and archaeology
[ ]	[x]	Animals and other organisms
[x]	[ ]	Human research participants
[x]	[ ]	Clinical data
[x]	[ ]	Dual use research of concern

Methods

n/a	Involved in the study	
[x]	[ ]	ChIP-seq
[ ]	[x]	Flow cytometry
[x]	[ ]	MRI-based neuroimaging

Antibodies

Antibodies used	Rabbit anti-GFP (ab32146,Abcam), anti-Rip3 (ab56164, Abcam), anti-p-Rip3 (ab222320, Abcam), mouse anti-β actin (ab6276, Abcam), goat anti-Rabbit IgG H&L (HRP) (ab6721, Abcam), goat anti-Mouse IgG H&L (HRP) (ab205719, Abcam), and mouse anti-GAPDH antibody (60004-1-Ig, Proteintech), anti-6X His tag® antibody (ab252883, Abcam), Donkey anti-mouse IgG H&L (Alexa Fluor 647) (A-31571, Thermo fisher).
Validation	Rabbit anti-GFP (ab32146,Abcam): this antibody was verified for WB by supplier(https://www.abcam.com/gfp-antibody-e385-ab32146.html).<br>Anti-Rip3 (ab56164, Abcam): this antibody was verified for WB by supplier(https://www.abcam.com/rip3-antibody-ab56164.html).<br>Anti-p-Rip3 (ab222320, Abcam): this antibody was verified for WB by supplier(https://www.abcam.com/rip3-phospho-t231--s232-antibody-epr19403-52-ab222320.html).<br>Mouse anti-β-actin (ab6276, Abcam):this antibody was verified for WB by supplier(https://www.abcam.com/beta-actin-antibody-ac-15-ab6276.html).<br>Goat anti-Rabbit IgG H&L (HRP) (ab6721, Abcam): this antibody was verified for WB by supplier(https://www.abcam.com/goat-rabbit-igg-hl-hrp-ab6721.html).<br>Anti-GAPDH antibody (60004-1-Ig, Proteintech): this antibody was verified for WB by supplier(http://www.ptgcn.com/Products/GAPDH-Antibody-60004-1-Ig.html).

	Anti-6X His tag® antibody (ab252883, Abcam):this antibody was verified for Immunohistochemical analysis by supplier(https://www.abcam.com/6x-his-tag-antibody-3d5-ab252883.html).<br>Donkey anti-mouse IgG H&L (Alexa Fluor 647) (A-31571, Thermo fisher):this antibody was verified for Immunohistochemical analysis by supplier(https://www.thermofisher.com/cn/zh/antibody/product/Donkey-anti-Mouse-IgG-H-L-Highly-Cross-Adsorbed-Secondary-Antibody-Polyclonal/A-31571)

Eukaryotic cell lines

Policy information about cell lines

Cell line source(s)	The HEK-293T human embryonic kidney cell line (CRL-3216) and mouse fibrosarcoma cell line L929 (CCL-1) , generously provided by Professor Jiahuai Han (Xiamen University),which were both obtained from ATCC.The MA104 embryonic rhesus monkey kidney cell line was obtained from ATCC (CRL2378.1).
Authentication	Cell lines were authenticated based on unique morphology and growth characteristics.
Mycoplasma contamination	We confirmed that HEK-293T cells ,L929 cells and MA-104 cells were mycoplasma-negative using Mycoplasma Detection Kit (InvivoGen).
Commonly misidentified lines<br>(See ICLAC register)	No commonly misidentified cell lines were used in the study.

Animals and other organisms

Policy information about studies involving animals; ARRIVE guidelines recommended for reporting animal research

Laboratory animals	Female BALB/c mice were supplied from the Shanghai SLAC Laboratory Animal Co., Ltd. The animals were housed in individual ventilated cages (IVCs) and allowed free access to food and water.The Six- to eight-week-old mice was used in the TNF-induced SIRS mouse model (including immunohistochemistry and ex vivo imaging); the five- to seven-week-old mice was used in APAP-induced acute liver failure model.
Wild animals	This study did not involve wild animals.
Field-collected samples	This study did not involve samples collected from fields.
Ethics oversight	All animal experiments were carried out in compliance with the regulations of the Animal Welfare and Ethics Committee at Xiamen University.

Note that full information on the approval of the study protocol must also be provided in the manuscript.

Flow Cytometry

Plots

Confirm that:

- [x] The axis labels state the marker and fluorochrome used (e.g. CD4-FITC).
- [x] The axis scales are clearly visible. Include numbers along axes only for bottom left plot of group (a 'group' is an analysis of identical markers).
- [x] All plots are contour plots with outliers or pseudocolor plots.
- [x] A numerical value for number of cells or percentage (with statistics) is provided.

Methodology

Sample preparation	Depending on the experiment, cells were grown to 80% confluence, harvested, and seeded at 60,000 cells/well in a 12 well plate and allowed to adhere overnight, then cells were treated as defined in the materials and methods sections.<br>Sample preparation for MFI of Split-GFP/full-length GFP analysis:<br>1.The Sample cells were washed three times with DMEM containing 10 U/mL heparin, the cells were detached.<br>2. Wash cells three times with cold PBS, then suspended with 500 µL cold PBS<br>3. Flow cytometry test.<br>Sample preparation for cell death analysis:<br>1.The Sample cells were washed three times with DMEM containing 10 U/mL heparin, the cells were detached;<br>2. Wash cells three times with cold PBS;<br>3. Suspended with 500 µL cold PBS containing 5 µg/mL PI., gently mix and incubate for 30 min-1 h at 4°C;<br>4. Flow cytometry test.
Instrument	Beckman-Coulter CyAn ADP*(PI) ;LSRFortessaX-20(Split-GFP/full-length GFP)
Software	Beckman Summit v4.3(PI);BD FACSDiva Software(Split-GFP/full-length GFP)
Cell population abundance	Cell population abundance was more than 5,000

Gating strategy

Gating strategy for Mean Fluorescence Intensity of split-GFP analysis
1. Open data in software and set the first scatter plot, X-axis select FSC, Y-axis select SSC. Then click right mouse button and select the Polygon region to set gate the cell population that need to be analyzed named R1.
2. Set the histogram, right click in R1 area and select set gate option, then double-click in this histogram make it showing only the cells in R1 will appear.
3. The Ruby signal is in PE channel, so the cell group in R1 is displayed in histogram, the GFP1-10 protein treated-cell sample is used to as the negative control. A bar is gating to select the cell of which florescence intensity of Ruby is higher than that of negative control, named R2.
4. Set the second scatter plot, right click in R2 area and select set gate option, then double-click in the second scatter plot make it showing only the cells in R2 will appear.
5. The experimental data GFP signal is in FITC channel, so the cell group in R2 is displayed in FITC/SSC scatter plot. A Polygon region is gating to circle the cell which florescence intensity of GFP is higher than that of negative control, named R3. At the same time, the overall Mean value in the histogram can be read out as the MFI value of the sample being tested.
Gating strategy for Mean Fluorescence Intensity of full-length GFP analysis
1. Open data in software and set the first scatter plot, X-axis select FSC, Y-axis select SSC. Then click right mouse button and select the Polygon region to set gate the cell population that need to be analyzed named R1.
2. The experimental data GFP signal is FITC channel, so the cell group in R1 is displayed in histogram, the GFP protein treated-cell sample is used to as the negative control. A bar is set to select the cell which florescence intensity of GFP is higher than that of negative control, named R2. At the same time, the overall Mean value in the histogram can be read out as the MFI value of the sample being tested.
Gating strategy for Mean Fluorescence Intensity of Cy-5 analysis
1. Open data in software and set the first scatter plot, X-axis select FSC, Y-axis select SSC. Then click right mouse button and select the Polygon region to set gate the cell population that need to be analyzed named R1.
2. The experimental data Cy-5 signal is APC channel, so the cell group in R1 is displayed in histogram, the Ppm1b-Cy5 treated-cell sample is used to as the negative control. A bar is set to select the cell which florescence intensity of Cy5 is higher than that of negative control, named R2. At the same time, the overall Mean value in the histogram can be read out as the MFI value of the sample being tested.
Gating strategy for cell survival analysis
1. Open data in software and set the first scatter plot, X-axis select FSC, Y-axis select SSC. Then click right mouse button and select the Ellipse region to set gate the cell population that need to be analyzed named R1.
2. Set the second scatter plot, right click in R1 area and select set gate option, then double-click in the second scatter plot make it showing only the cells in R1 will appear.
3. The experimental data PI signal is in PE channel, so the cell group in R1 is displayed in PE/SSC scatter plot, the untreated cell sample is used to as the negative control. A Polygon region is gating to circle the cell which cell ratio of PI is higher than that of negative control, i.e. necrosis cell, named R2.

☒ Tick this box to confirm that a figure exemplifying the gating strategy is provided in the Supplementary Information.
